# Expert opinion on the treatment of patients with chronic hepatitis C

**DOI:** 10.1111/j.1365-2893.2008.01012.x

**Published:** 2009-02

**Authors:** S Zeuzem, T Berg, B Moeller, H Hinrichsen, S Mauss, H Wedemeyer, C Sarrazin, D Hueppe, E Zehnter, M P Manns

**Affiliations:** 1 Zentrum der Inneren Medizin, Klinikum der Johann Wolfgang Goethe-UniversitätFrankfurt, Germany; 2 Medizinische Klinik m. S. Hepatologie und Gastroenterologie Charité, Campus Virchow-Klinikum, Universitätsmedizin BerlinBerlin, Germany; 3 Hepatologische SchwerpunktpraxisBerlin, Germany; 4 GemeinschaftspraxisKiel, Germany; 5 Internistische PraxisDüsseldorf, Germany; 6 Medizinische Hochschule Hannover, Abt. Gastroenterologie und Hepatologie, Zentrum Innere MedizinHannover, Germany; 7 Gastroenterologische Gemeinschaftspraxis Herne, Ärztehaus am Evangelischen Krankenhaus HerneHerne, Germany; 8 Internistische PraxisDortmund, Germany

**Keywords:** hepatitis C, pegylated interferon alfa, ribavirin, treatment, virological response

## Abstract

The current preferred treatment for patients with hepatitis C virus (HCV) is combination therapy consisting of pegylated interferon alfa and ribavirin (RBV) for 24–48 weeks. Although this approach appears to be highly effective for patients with HCV genotypes 2 or 3, who have a sustained virological response (SVR) of approximately 80%, the treatment algorithm is less effective for patients with HCV genotype 1, as these patients have SVR rates of just 40–50%. In order to improve treatment outcomes, this article explores potential approaches for the optimization of treatment for patients with HCV genotype 1: considering shorter treatment periods for patients with a rapid virological response (RVR), increasing treatment periods for slow responders, and increasing RBV dose are all suggestions. Results from clinical trials suggest that approximately 20% of the HCV genotype 1-infected population are slow responders, and around 15% of all HCV genotype-1 infected patients could benefit from a shorter treatment duration without compromising the SVR rate. Interest has also focused on whether treatment duration could be individualized in some patients with genotype 2 and 3 infection. Here all the findings from recent studies are translated into practical advice, to help practitioners make evidence-based treatment decisions in everyday clinical practice. Although there are areas where currently available data do not provide conclusive evidence to suggest amending treatment approaches, there is clearly potential for individualized treatment in all aspects of hepatitis treatment in the future.

## Introduction

Current treatment algorithms result in rates of sustained virological response (SVR) of ∼80% in patients infected with HCV genotypes 2 or 3, suggesting that some of the primary challenges in the management of chronic hepatitis C (CHC) have now been resolved. However, in patients infected with HCV genotype 1, the standard combination treatment of 48 weeks of pegylated interferon alfa (peginterferon) and ribavirin (RBV) results in SVR rates of only 40–50% [1,2], with higher rates following 48 weeks rather than with 24 weeks of treatment (51%*vs* 41%, respectively) [3]. Emerging data suggest that treatment duration may be shortened or lengthened depending on baseline viral load and virological response at week 4 and/or week 12. This paper considers these results and their implications for treatment optimization and suggests how this latest research can be translated into everyday clinical practice.

## Issues Under Consideration

Principal considerations for treatment of CHC include dose and duration of antiviral therapy (along with related costs), quantification of baseline HCV RNA levels, the definition of response during the early stages and at the end of treatment, as well as the duration of the post-treatment follow-up period. In addition, there remain a number of areas of uncertainty that have also to be taken into consideration, such as the variation in baseline viral load, monitoring time points and the ‘time window’ within which monitoring needs to take place.

### Current treatment algorithm for treatment of patients with HCV

**Fig. 1:**
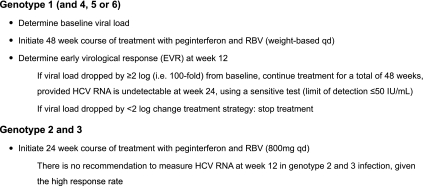
Overview of current treatment guidelines (based on references [4–6,12,13]).

Current treatment recommendations for patients chronically infected with HCV are shown in Fig. 1 [4–6]. Briefly, patients with genotype 2 or 3 infection are more responsive to the current standard of care of peginterferon plus RBV than those with genotype 1 or genotype 4 infection. The rates of SVR for genotype 2 or 3 infection are similar in patients treated for 24 or 48 weeks; thus, for these patients 24-week treatment is generally considered appropriate. For patients infected with HCV genotype 1, the recommended treatment duration is 48 weeks of peginterferon with RBV. While standard doses for peginterferon alfa-2a (180 μg, qw) and peginterferon alfa-2b (1.5 μg/kg, qw) are well established, different recommendations exist for RBV dose according to HCV genotype and type of peginterferon [7,8]. It appears that lower doses of RBV are required for treatment of patients infected with HCV genotype 2 or 3 than for genotypes 1 or 4 [9,10]. For the standard duration of treatment of HCV genotype 1 and 4 infection, weight-based RBV doses of 800–1200 mg, qd, or up to 1400 mg for patients above 105 kg, are recommended, while no additional benefit of RBV doses higher than 800 mg in HCV genotype 2 and 3 infection was observed in several studies [3,11]. Available data for patients infected with genotype 5 or 6 are limited; therefore, combination treatment with 1000/1200 mg, qd, RBV for 48 weeks is currently recommended.

### Determination and monitoring of viral load

The decision on whether to continue or stop therapy should primarily be based on the level of HCV RNA during treatment. Therefore, it is necessary to measure viral load accurately. Important aspects to consider in this respect are the natural fluctuations in viral load during infection, as well as intra-assay (within an individual test) and inter-assay (between different tests) variability. Currently available commercial assays vary considerably in their dynamic ranges of quantification (Table 1). Despite the introduction of international units per mL (IU/mL) for reporting viral load, discrepancies may occur when patients are monitored using different types of assay [14–19]. For example, rules for early discontinuation at week 12 and 24, as well as rules for determination of treatment duration [baseline viral load, RVR, complete early viral response (cEVR)], were established mainly with standard RT-PCR based assays, which have since been replaced by real-time PCR-based assays with higher sensitivity and broader dynamic range of linear HCV RNA quantification. The differences between commercial HCV RNA assays have been well documented in several studies [15–19], with the majority of studies showing an intra-assay variability of approx. 0.2 log. Generally, comparisons between Amplicor Monitor and CAP/CTM yielded comparable results (±0.2 log), whereas comparisons between bDNA and Abbott real-time HCV on the one hand and CAP/CTM on the other showed a difference of 0.5–0.7 log. Additionally, HCV RNA viral load decline assessed during antiviral therapy can give different results, regardless of the use of IUs. False-positive and false-negative results, as well as variations in the HCV RNA level of up to 2 log_10_ IU, have been observed, which may well have an impact on the management of patients, particularly if treatment decisions are made using a single HCV RNA assessment [15,16,19]. Practitioners should be careful not to attach undue clinical significance to small changes (<0.5 log_10_) in serum HCV RNA level. The clinical relevance of serial HCV viral level measurements in a patient is dependent on continuous use of the specific quantitative assay employed in the initial determination of the viral level. This may imply repeated testing in some cases; but these extra costs may be justified if they affect treatment management decisions.

**Table 1:** Detection limits and range of linear quantification for HCV RNA tests [20]

		Dynamic range of linear quantification IU/mL	
Test	Detection limit (cut-off) IU/mL	Lower limit	Upper limit
Qualitative assays			
Versant qualitative assay (Siemens, Eschborn, Germany)	5–10	NA	NA
Cobas Amplicor v2.0 (Roche, Mannheim, Germany)	50	NA	NA
Quantitative assays			
Abbott Real Time	10	12	100 000 000
Cobas TaqMan real-time PCR assay (Roche)	10	43	69 000 000
Cobas Amplicor Monitor v2.0 (Roche)	600	600	500 000
Versant HCV RNA 3.0 (Bayer)	615	615	7700 000

## Genotype 1

### Week 12 stopping rule for patients with HCV genotype 1

The current week 12 stopping rule recommends that patients without a ≥2 log_10_ drop in viral load compared to baseline (between 19% and 29% of patients with genotype 1 infection) discontinue therapy since the likelihood of achieving SVR with continued treatment is small; the negative predictive value is almost 100% [21,22]. Over-treatment of patients who have an extremely low chance of achieving SVR is thus avoided and valuable resources can be reserved for patients with a higher chance of treatment success [23]. Week 12 monitoring should be carried out as close as possible to the week 12 time point, ideally ±5 days, using a test with high sensitivity and wide dynamic range. Whether the 2 log_10_ drop represents the most accurate cut-off level for the decision on treatment termination or proceeding remains to be determined in prospective clinical studies. It is likely that with greater use of more sensitive assays with a broader range of linear quantification (e.g. real-time PCR assays), this parameter may be refined/adjusted in the near future. It may also be the case that new drugs currently in development will require different threshold levels and/or stopping rules based on their different modes of action, although this remains to be seen.

### Assessment at week 24 in patients with HCV genotype 1

If, at week 12, HCV RNA remains detectable but the viral load has dropped by at least 2 log_10_ (i.e. 100-fold) from baseline, treatment should be continued for the full 48-week course. However, if the patient remains HCV RNA positive at week 24, it is unlikely that an SVR will be achieved (negative predictive value 98–100%), [2,21,24], and, unless the patient is considered at high risk due to rapidly progressing fibrosis, treatment termination at week 24 can be considered. Studies are ongoing to determine whether patients may derive some benefit from treatment with peginterferon monotherapy, despite a lack of virological response. These include the COPILOT study comparing colchicine with low-dose peginterferon alfa-2b [25,26], which showed both high rates of premature discontinuation of therapy and that maintenance therapy with peginterferon was associated with improved disease free survival almost exclusively in patients with portal hypertension, and the EPIC3 program with peginterferon alfa-2b [27]. Recent results from the HALT-C trial [28], which investigated the effect of treating non-responders with peginterferon alfa-2a and RBV concluded overall that long-term therapy with peginterferon did not reduce the rate of disease progression and so do not support maintenance therapy in patients with HCV and advanced hepatic fibrosis who are prior non-responders. Interestingly, a significant decline in clinical outcomes was observed in patients with chronic HCV and advanced fibrosis or cirrhosis who achieved a profound decline in HCV RNA, defined as >4 log and/or undetectable with subsequent breakthrough or relapse, suggesting that a small subgroup of patients may benefit [29]. Unless results of the ongoing studies provide additional guidance, continued treatment of patients cannot be recommended.

### Recommendations for optimizing treatment in patients with HCV genotype 1

#### Shorter treatment for patients with a rapid virological response

**Fig. 2:**
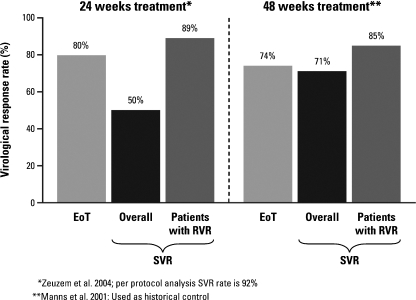
Rapid virological response predicts sustained virological response in HCV-1 infected patients with low baseline viral load (≤ 600 000 IU/mL).

The current 48-week treatment duration, recommended for HCV genotype 1-infected patients, may potentially result in the over-treatment of some genotype 1-infected patients who are more likely to achieve SVR, i.e. patients with low viral load before treatment and rapid virological response (RVR) at week 4. Clearly it is desirable to expose patients to the shortest possible treatment duration – without compromising efficacy – in order to minimize the likelihood of adverse events and reduce costs. Hadziyannis *et al.* found that more than one third of individuals with HCV genotype 1 who were randomized to 24 weeks of therapy with pegylated IFNα-2a plus RBV achieved SVR [3]. Moreover, patients infected with HCV genotype 1 who became HCV RNA-negative by week 4, i.e. patients with RVR, were more likely to achieve SVR than those who did not become HCV RNA negative until week 12 [22]. A recent prospective trial demonstrated that patients with low baseline HCV RNA levels (≤600 000 IU/mL) and an RVR achieve an SVR rate of up to 90% (Fig. 2) [30]. Jensen *et al.* observed that almost a quarter (22.6%) of HCV genotype 1 patients treated with peginterferon plus RBV achieved RVR [31]. Of these patients, 89% showed SVR after treatment duration of only 24 weeks. Both pegylated interferons have recently been approved in the EU for shortened treatment duration of 24 weeks for HCV genotype 1 patients with low-viral load (LVL) (defined as <800 000 IU/mL for peginterferon alfa-2a and <600 000 IU/mL for peginterferon alfa-2b) and RVR [7,8]. To assure accurate determination of baseline viral load in cases with HCV RNA concentrations between 400 000 and 1 million IU/mL, physicians should consider performing two measurements using the same technique, from samples taken at least 4 weeks apart. Whether 10 or 50 IU is the most appropriate cut-off for determining RVR remains unclear, however, and is under investigation. Recently, Sarrazin *et al.* compared clinical outcomes for large cohorts of patients whose serum samples were analysed using both the COBAS TaqMan™ (detection limit approximately 10 IU/mL) and COBAS Amplicor™ (detection limit <50 IU/mL) assays. In this study, RVR rates and subsequent SVR rates were similar when RVR was defined as undetectable of below 15 IU/mL by the COBAS TaqMan assay in comparison with undetectable (<50 IU/mL) by the COBAS Amplicor assay, implying that HCV RNA levels rapidly decline not only to below 50 IU/mL but also below 15 IU/mL in patients achieving an RVR [15]. Interestingly, relapse rates were consistently lower in patients with undetectable HCV RNA at week 4 by COBAS TaqMan™ compared with COBAS Amplicor™, although the full significance of this remains to be established [15].

Patients should not be considered for shorter treatment duration if they have a baseline viral load above 600–800 000 IU/mL and/or have cirrhosis, are co-infected with HIV, or are immunosuppressed. Other factors influencing virological response that may also be considered include metabolic syndrome, insulin resistance and extensive steatosis. Zeuzem *et al.* demonstrated that the efficacy of peginterferon alfa-2a plus RBV is comparable between patients with genotype 1 infection and persistently normal alanine-aminotransferase (ALT) and those with elevated ALT levels [32]. However, SVR rates were significantly lower in those patients with persistently normal ALT treated for 24 weeks compared with 48 weeks (13%*vs* 40%, respectively), which also suggests that such patients may not be suitable candidates for shorter therapy. As this study of patients with persistently normal ALT did not include evaluation of RVR, it was not possible to identify a potential patient subgroup within this population (e.g. low viral load and/or RVR) who might benefit from shorter treatment.

## Determining Pre-Treatment Viral Loads and Defining Low *vs* High-Viral Loads

The definition and differentiation between low and high viral loads is still under discussion. Historically, pre-treatment viral load was classified as ‘high’ or ‘low’ using a cut-off of 2 × 10^6^ copies/mL, based on data generated using conventional interferon-based regimens or pegylated interferon monotherapy [33,34]. When HCV RNA assays were standardized, conversion of copies/mL to IU/mL according to the WHO standard gave varying results depending on the assay used; 800 000 IU/mL has been recommended as the decision threshold for high versus low viraemia [35]. However, recent data suggest that a baseline level of 400 000 IU/mL is the most effective cut-off for a high or low probability to achieve SVR in genotype 1-infected patients [36,37]. This level was confirmed in a large ‘real-life’ experience study [38] and in a further study by Martinot-Peignoux and colleagues, with the caveat that it should be applied to treatment-naïve patients only [39]. In a recent study, pre-treatment HCV-RNA levels of 250 000 IU/mL best discriminated between genotype 1-infected patients with or without SVR after 24 weeks of therapy in patients with low pre-treatment viral load [37]. Whether a single cut-off level for pre-treatment viraemia is sufficient or whether several ranges of pre-treatment HCV RNA levels might allow for individualized treatment duration remains to be elucidated. Furthermore, cut-offs for low or high baseline HCV RNA concentration were established mainly on the basis of standard RT-PCR and bDNA assays and re-definition by the currently used real-time PCR-based assays is required. According to current data, treatment duration of 24 weeks in genotype-1 infected patients should be strongly considered for patients who achieve RVR and have a baseline viral load below 800 000 IU/mL.

## Determining RVR At Week 4

Patients who are considered for shortened treatment duration must be tested at week 4 for RVR (i.e. no HCV RNA detectable) using a highly sensitive method (limit of detection ≤50 IU/mL) [15]. The week 4 value should be measured as close as possible to day 28 of therapy, i.e. between the fourth and fifth injection of peginterferon. Patients without assessment of RVR should not be considered as candidates for shortened therapy duration.

Monitoring is an important feature in the management of CHC; not only to document treatment success, but also as an indicator of compliance and adherence. Patients with RVR at week 4 should be tested again at week 12 (±5 days). The probability that the PCR test is negative at week 4 but positive at week 12 is low; only 1 of 22 patients who experienced virological breakthrough prior to week 24 had an RVR [40].

### Optimizing response by reducing relapse rates in patients with HCV genotype 1

A patient with virological relapse is one who achieved an end-of-treatment (EOT) response but who failed to achieve an SVR. Relapse has been reported to occur at similar rates for patients treated with peginterferon alfa-2a and -2b (18% and 19%, respectively) who were treated for 48 weeks according to the standard treatment algorithm [1,2]. The IDEAL study, which investigates response to peginterferon alfa-2a and two different doses of peginterferon alfa-2b with RBV in patients with genotype 1 CHC, is also addressing this issue [41]. Intensification of treatment is a possible approach to reduce the incidence of relapse. IDEAL is accepted as late-breaker at EASL 2008.

## Increased Dose of Ribavirin

Recent studies suggest that high-dose RBV in combination with pegylated interferon can improve response in genotype 1-infected patients. Lindahl *et al.* used an individualized dosing regimen based largely on renal function, in an attempt to achieve >15 μmol/L steady-state RBV concentration in 10 treatment-naïve patients [42]. After initial dose adjustments, the mean dose of RBV was 2540 mg, qd (range 1600–3600 mg, qd) and the mean RBV concentration achieved was 14.7 μm (range 7.8–22.0 μm) at weeks 24–48. Nine of 10 patients achieved SVR following treatment of up to 48 weeks duration, but with more frequent and severe side effects, in particular anaemia. All patients required erythropoietin at some time during treatment.

A recent study by Fried *et al.* demonstrated an improvement in SVR in genotype 1-infected patients with body weight >85 kg treated with a higher dose of RBV, especially in conjunction with a higher dose of peginterferon [43]. Patients treated with 270 μg peginterferon alfa-2a and 1600 mg, qd, RBV showed an SVR of 47% compared with 28% in patients treated with the standard dosing regimen. This improvement was driven mainly by a marked reduction in relapse in the high-dose group compared with the standard-dose group (19%*vs* 40%, respectively). However, the use of a higher dose regimen was associated with an increased rate of haematological adverse events. More recently, in a prospective, open-label, randomized, controlled pilot study comparing 48 weeks of treatment with peginterferon plus standard weight-based RBV with or without erythropoietin (groups 1 and 2), and peginterferon plus higher weight-based RBV plus erythropoietin (group 3), SVR was significantly greater (*P*<0.05) in group 3 patients (49%) due to a significant decline in relapse rate [44]. Overall, the results of these studies provide encouraging data regarding the possibility of optimizing treatment regimens for patients with more difficult to treat disease.

## Extending Treatment Duration for Slow Virological Responders

Evidence from three randomized clinical trials support the case for extending treatment duration beyond 48 weeks in HCV genotype 1 patients with a slow virological response, i.e. HCV RNA > 50 IU/mL at week 12 but undetectable (<50 IU/mL) at week 24 [45–47]. Berg *et al.* randomized patients to 48 or 72 weeks of treatment with peginterferon alfa-2a (180 μg, qw) plus RBV (800 mg, qd) and analysed the resulting SVR and relapse rates [45]. Extended treatment of 72 weeks did not increase the SVR rate in the intent-to-treat population; which suggests that it is inappropriate to extend treatment for all genotype 1-infected patients. However, the study demonstrated that identifying patients with and without virological response at week 12 using a sensitive molecular test (50 IU/mL) could facilitate the decision on therapy duration for each patient on an individual basis. Patients who remained HCV RNA positive at week 12 had significantly higher SVR rates when treated for 72 rather than 48 weeks (29%*vs* 17%; *P*=0.04). The greatest benefit from extended treatment duration (72 weeks) was observed in patients with detectable HCV RNA, but with levels below 6000 IU/mL, at week 12. The frequency and intensity of adverse events was similar in the 48- and 72-week treatment groups, suggesting that extended treatment can be manageable in terms of tolerability. Sanchez-Tapias *et al.* randomized patients without RVR (i.e., HCV RNA > 50 IU/mL at week 4) to treatment with pegylated interferon alfa-2a (180 μg, qw) and RBV (800 mg, qd) for 48 or 72 weeks [46]. Extending treatment to 72 weeks significantly increased the SVR rate compared with the standard 48 weeks of therapy (45%*vs* 32%, respectively; *P*=0.01). In genotype 1-infected patients, this effect was particularly evident, with 44% of patients who received 72 weeks of treatment achieving SVR compared with 28% of patients who were treated for 48 weeks (*P*=0.003). The incidence of adverse events was similar between the two groups, although treatment discontinuation was significantly more frequent in the 72-week group (36%*vs* 18%; *P*=0.0004). A retrospective analysis of patients from the European-based trials, including that of Berg *et al.*, demonstrated that patients without RVR but achieving subsequent early viral response (EVR) (>2 log_10_ HCV RNA decrease), benefited from extending treatment duration and achieved a higher SVR rate (77% after 72 weeks *vs* 31% after 48 weeks [48].

Pearlman *et al.* examined the effect of longer treatment duration with pegylated interferon alfa-2b plus weight-based RBV in patients infected with HCV genotype 1 who met EVR criteria at week 12 (>2 log_10_ drop in baseline HCV RNA), but who had detectable HCV RNA at week 12 and became HCV RNA-negative at week 24 [47]. This group of ‘slow’ viral responders was then treated for either 48 or 72 weeks. Results showed a 39% SVR in the 72-week arm compared with 18% SVR in the 48-week arm; treatment extension did not seem to result in an increase in dose reductions of RBV or discontinuations. Taken together, the available data suggest that longer duration of therapy improves sustained response rates in ‘slow’ virological responders.

### Proportion of HCV genotype 1-infected patients who could be considered for shortened or extended therapy duration

**Fig. 3:**
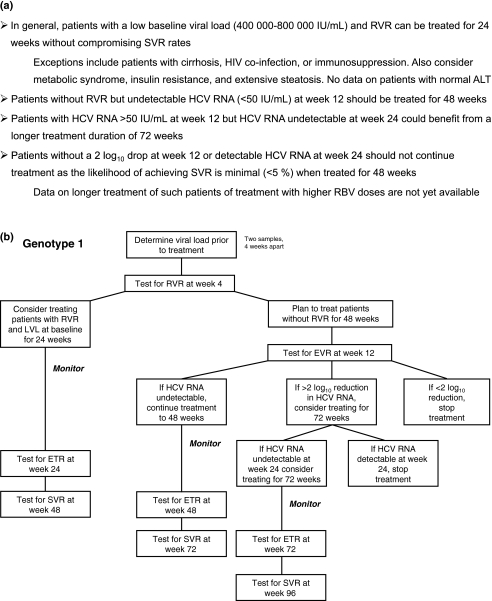
(a) Proposed treatment algorithm for patients with HCV genotype 1 based on response at weeks 4 (RVR), 12 (EVR) and 24. (b) Guidance for treatment and monitoring of response to peginterferon/RBV combination therapy in patients infected with HCV genotype 1. EVR, early viral response; RVR, rapid viral response; SVR, sustained viral response; PCR, polymerase chain reaction; ETR, end-of-treatment response.

An extended treatment duration of 72 weeks can be considered in ‘slow’ virological responders, defined as patients who are HCV RNA positive at week 12 but become undetectable at week 24. These patients comprise approximately 20% of the HCV genotype 1-infected population, a not insubstantial proportion [45]. Similarly, around 15% of all HCV genotype-1 infected patients could benefit from a shorter treatment duration without compromising the SVR rate; again constituting a clinically relevant proportion of patients [30]. A summary of the recommendations for optimizing treatment in patients with HCV genotype 1 is given in Fig. 3.

## Genotypes 2 and 3

Interest has also focused on whether treatment duration could be individualized in some patients with genotype 2 and 3 infection; i.e. shortened due to the overall high rate of SVR (∼80%) achieved with the standard 24 weeks of treatment, or prolonged in slow responders.

### Recommendations for optimizing treatment in patients with HCV genotypes 2 and 3

#### Shorter treatment for patients with a rapid virological response

A number of studies have investigated whether it might be possible to reduce treatment duration in some patients with chronic HCV genotypes 2 or 3 infection based on RVR. Several small studies have demonstrated comparable SVR rates after 16 weeks and 24 weeks treatment in patients with either genotype 2 (Table 2) or 3 (Table 3) infection who achieve an RVR [49–52]. However, in the large-scale randomized, multinational ACCELERATE study, in which a lower dose of RBV was used, overall SVR rates were lower following 16 weeks of peginterferon plus RBV compared with 24 weeks treatment in genotype 2 and 3 patients, although this difference only reached significance in genotype 2 patients [9]. Among the patients with an RVR, SVR rates were significantly higher in the 24-week group than in the 16-week group, both overall (85%*vs* 79%, *P*<0.001) and within each genotype group, although patients who achieved an RVR were more likely to achieve an SVR overall [9]. Overall, the significant difference seen in SVR rates was found to reflect a significantly higher relapse rate in the 16-week group (31%) compared with the 24-week group (18%; *P*<0.001); shorter treatment duration was associated with a significantly higher risk of relapse in both genotype 2 and 3 patients [9].

**Table 3:** Overview of short-term treatment versus standard (24-week) treatment in patients (pts) with HCV genotype 3 infection

Ref	Duration (weeks)	Ribavirin dose (mg/day)	SVR in pts with RVR following shorter duration therapy (%)	SVR in pts with RVR following standard duration therapy (%)	SVR in RVR-pts with low *vs* high baseline viraemia following shorter duration therapy	Relapse rate following shorter *vs* standard duration in RVR-pts
54	14*	800–1400	89	N/A	98%*vs* 79%†	11%*vs* N/A
50‡	12*	1000–1200	77	100	N/A	4%*vs* N/A
51	16	800–1200	76	75	93%*vs* 54%*§	N/A
9‡	16	800	80	85	No data	No data
55	14	800–1400	84	92	80%*vs* 86%**	16%*vs* 8%

* Included only patients with RVR.

‡ Patients randomized before treatment.

† HCV RNA ≤ 600 000 IU/mL *vs* >600 000 IU/mL.

§ HCV RNA ≤ 800 000 IU/mL *vs* >800 000 IU/mL.

** HCV RNA ≤ 400 000 IU/mL *vs* >40 000 IU/mL.

**Table 2:** Overview of short-term treatment versus standard (24-week) treatment in patients (pts) with HCV genotype 2 infection

Ref	Duration (weeks)	Ribavirin dose (mg/day)	SVR in pts with RVR following shorter duration therapy (%)	SVR in pts with RVR following standard duration therapy (%)	SVR in RVR-pts with low *vs* high baseline viraemia following shorter duration therapy	Relapse rate following shorter *vs* standard duration in RVR-pts
54	14*	800–1400	91	N/A	92%*vs* 88%*†	10%*vs* N/A
50‡	12*	1000–1200	87	89	N/A	9%*vs* N/A
51	16	800–1200	95	95	100%*vs* 93%*§	N/A
53‡	16	800–1400	100	98	No data	0%*vs* 2%
9‡	16	800	78	85	No data	No data
55	14*	800–1400	93	97	100%*vs* 90%**	7%*vs* 3%

* Included only patients with RVR.

‡ Patients randomized before treatment.

† HCV RNA ≤ 600 000 IU/mL *vs* >600 000 IU/mL.

§ HCV RNA ≤ 800 000 IU/mL *vs* >800 000 IU/mL.

** HCV RNA ≤ 400 000 IU/mL *vs* > 400 000 IU/mL.

There is some evidence to suggest that genotype 2 and 3 may respond differently to treatment; overall SVR rates tend to be somewhat lower for genotype 3 patients who do not achieve an RVR compared with genotype 2 patients who do not achieve RVR, and also after shorter treatment duration in patients who do achieve RVR [9,50,51,53,54]. Whether genotype 3-infected individuals should not therefore be considered for shorter duration therapy requires further investigation.

Baseline HCV RNA levels also influence SVR rates and patients with low pre-treatment serum HCV RNA levels and RVR have been reported to respond equally well to both 16 and 24 weeks of therapy (SVR rates of 82–100% and 81–100%, respectively) [9,49,50,53]. It is possible, therefore, that these patients may be considered for shorter treatment duration.

Genotype 2 and 3 infected patients with severe fibrosis are less likely to achieve either RVR or SVR, and to relapse more frequently following 12–14 weeks of antiviral therapy [9,49,51,53]. Andriulli *et al.* found that patients with low pre-treatment ALT levels were also found to relapse more frequently following shorter treatment duration (14% after 12–14 weeks *vs* 2% after 24 weeks; *P*=0.04) [51]. These findings suggest that patients with severe fibrosis or normal pre-treatment ALT levels are most likely unsuitable candidates for short-term treatment, but prospective studies are needed to confirm these observations.

#### Optimizing response by reducing relapse rates in patients with HCV genotypes 2 and 3

There is evidence that patients with HCV genotype 2 or 3 and higher baseline viral load have lower rates of SVR and higher relapse rates after 24 weeks of treatment than those with lower HCV RNA baseline levels [9,32,50,55], and that, in patients without RVR, the lowest rates of relapse are obtained with 48 weeks of treatment and a higher RBV dose [11]. Whether increasing treatment duration would help reduce relapse rates in patients with high baseline viral load requires further evaluation.

Although patients with genotypes 2 and 3 are generally considered to respond similarly to treatment, there is also some evidence to suggest that genotype 3 patients have lower SVR rates and subsequently higher relapse rates than genotype 2 patients [32]. An analysis of data from the WIN-R trial of peginterferon alfa-2b also demonstrated higher SVR rates and lower relapse rates in genotype 2 infected patients compared with genotype 3 (72%*vs* 63%, and 5%*vs 10%, respectively*) [56]. It is possible that genotype 3-infected patients would benefit from longer treatment duration and/or higher RBV dose compared with genotype 2-infected patients; however current data supporting this comes predominantly from retrospective analyses and requires evaluation in prospective clinical trials.

A recent study suggests that patients infected with genotype 3 who have cirrhosis are 10 times more likely to relapse following treatment with conventional or peginterferon plus RBV than those without cirrhosis [57], a finding consistent with the results from the trial by Hadziyannis *et al.* and described below [3]. If data from cirrhotic patients infected with HCV genotype 1 are extrapolated to those with other genotypes, it is likely that longer treatment duration may be beneficial in reducing relapse in genotype 2 and 3 patients with cirrhosis. However, this remains to be established in prospective clinical studies.

#### Proportion of HCV genotype 2 and 3-infected patients who could be considered for shortened or extended therapy duration

**Fig. 4:**
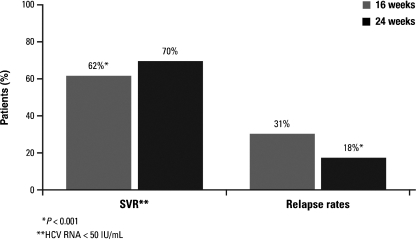
Overview of the ACCELERATE data: 16 *vs* 24 weeks. SVR: sustained viral response.

In general, patients infected with HCV genotypes 2 or 3 should not be routinely treated for less than the currently recommended 24 weeks to avoid an increased risk of virologic relapse. However, patients with a low pre-treatment viral load (≤400 000 IU/mL) and an RVR (as determined by a highly sensitive assay) have the highest probability of achieving an SVR with 16 weeks of therapy (Fig. 4). Such a regimen may be a reasonable option for these patients, especially if tolerability of longer treatment may be a concern. The cut-off for low-viral load in patients with genotypes 2 and 3 based on the ACCELERATE data is ≤400 000 IU/mL [9]. As with genotype 1, baseline viral load should be determined in two samples, taken at least 4 weeks apart.

Shortening of treatment duration should not be considered for patients with cirrhosis, persistently normal ALT values or co-infection with HBV or immunocompromised patients such as those with HIV infection or those who have undergone liver transplantation.

There may be patient subgroups with genotype 2 or 3 infection that might benefit from extended treatment duration to reduce relapse rates. Results from the trial by Hadziyannis *et al.* found evidence for reduced relapse rates in genotype 2 and 3 pts with advanced fibrosis/cirrhosis as well as high baseline viral load when treated for 48 weeks in comparison with 24 weeks [3].

**Fig. 5:**
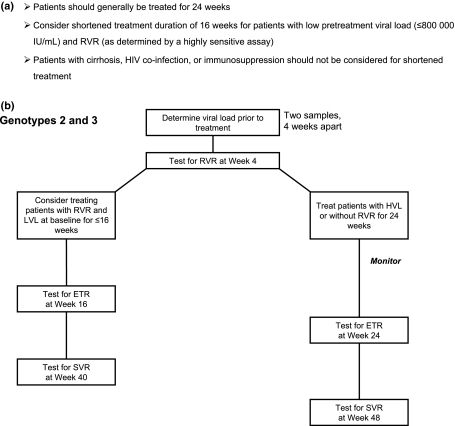
(a) Proposed treatment algorithm for patients with HCV genotype 2 or 3 based on response at week 4 (RVR). (b) Guidance for treatment and monitoring of response to peginterferon/RBV combination therapy in patients infected with HCV genotypes 2 or 3. EVR, early viral response; RVR, rapid viral response; SVR, sustained viral response; PCR, polymerase chain reaction; ETR, end-of-treatment response; LVL, low viral load; HVL, high viral load.

However, due to insufficient data from prospective clinical trials, there is currently not enough evidence for such recommendations. It is possible that a higher, weight-based dose of RBV may balance the increased risk of relapse associated with a shorter treatment duration, but this remains to be proven. An overview of the proposed treatment strategy for patients with genotypes 2 and 3 is given in Fig. 5.

## Additional Factors to Consider

### Consideration of ribavirin dose

RBV monotherapy does not induce a significant antiviral response in patients with CHC, but in combination with interferon, RBV markedly improves ETR response, reduces relapse rates and improves SVR rates. A number of mechanisms including direct inhibition of RNA replication, immunomodulation, inhibition of inosine monophosphate dehydrogenase, and enhanced viral mutagenesis have been proposed to explain the action of RBV in CHC as reviewed by Dixit *et al.* but its overall mode of action remains to be fully elucidated [58]. The main serious adverse event associated with the use of RBV is dose-dependent haemolytic anaemia. The optimal target dose for RBV is not well established.

A recent publication supports the individualization of RBV dosing according to HCV genotype and bodyweight, and highlights a number of clinical variables that influence the likelihood of SVR in contrast to the occurrence of anaemia [10]. The percentage of patients with SVR increased from 40% to 50% when RBV dose was increased from 12 to 16 mg/kg in patients with genotype 1 infection, but was much less influenced by RBV dose in genotype 2 and 3 patients [10]. A higher apparent clearance of RBV, older age and cirrhosis had a negative impact on achieving an SVR. Gender and RBV dose/kg were the most important prognostic factors for anaemia. However, as anaemia is not a universal risk in all treated patients, the initial high dosing strategy of 1000 or 1200 mg, qd, according to bodyweight appears to be appropriate. For heavier patients, RBV doses >1200 mg, qd, may be initiated as they are likely to be associated with additional efficacy and a manageable anaemia risk (provided that the dose does not greatly exceed 15 mg/kg/day) [10]. As discussed above, Lindahl *et al.* demonstrated in a small pilot study that administration of ultra high-dose RBV (ranging from 1600–3600 mg, qd) in genotype 1 patients according to an individualized schedule is feasible but is associated with more frequent and serious side effects such as anaemia [42]. In this albeit small study, nine of 10 patients achieved an SVR; however, two of 10 patients required blood transfusion for haemoglobin levels <8.0 g/dL and all patients required treatment with erythropoietin (range 9000 to 30 000 IU/week) and oral iron supplements. However, recommendations cannot be made on observations made on such limited patient numbers.

Retrospective analyses of the original study by Hadziyannis *et al.* showed that in patients without RVR, the lowest rates of relapse were obtained with 48 weeks of treatment and a higher RBV dose [11]. In contrast, treatment duration and RBV dose did not influence SVR in patients with RVR. For genotype 2 and 3 infected patients, higher RBV doses do not improve SVR or relapse rate in patients with RVR on the standard 800 mg, qd, dose [11]. However, for the minority of patients who do not achieve RVR, it is possible that increased RBV dose may improve treatment outcome, although sufficient data are not yet available to make a clear recommendation [11]. A study by Ferenci *et al.* suggests that, in genotype 2 and 3 infected patients treated with a standard fixed dose of 800 mg, qd, RBV, SVR was greater in patients who were exposed to the highest mean dose of RBV based on body weight [59]. These findings are consistent with a retrospective analysis of data from the peginterferon alfa-2b registration trial [2]. Nonetheless, whether increasing RBV exposure in heavier patients will improve SVR remains uncertain.

A number of studies have shown that erythropoietin can be used to improve quality of life, maintain RBV dose and subsequently improve adherence [60–62]. Although erythropoietin may have a role in the management of RBV-related anaemia, a recent study by Shiffman *et al.* failed to show an improvement in SVR in genotype 1-infected patients given epoetin alpha at the initiation of therapy to maintain haemoglobin levels between 12 and 15 g/dL [9]. This was a three arm, prospective, open-label, randomized, controlled pilot study comparing 48 weeks of treatment with peginterferon plus standard weight-based RBV with or without erythropoietin (groups 1 and 2), and peginterferon plus higher weight-based RBV plus erythropoietin (group 3). A significantly smaller percentage of group 2 patients had a decline in haemoglobin to less than 10 g/dL (9%*vs* 34%; *P*<0.05) and required that the RBV dose be reduced (10%*vs* 40%; *P*<0.05) compared to group 1 patients. Despite this, SVR was similar in these groups (19–29%). SVR was significantly greater (*P*<0.05) in group 3 patients (49%) due to a significant decline in relapse rate (Table 4).

**Table 4:** Virologic response in patients treated for 48 weeks with standard or higher weight-based doses of ribavirin

	PEG IFN α-2a + WBR	PEG IFN α-2b + WBR + EPO	PEG IFN α-2a + HWBR + EPO
ITT population (*n*)	48	49	49
Mean RBV dose (mg/day)	1016 ± 170	1102 ± 174	1224 ± 175
Dose reduction (%)	40	10	31
RVR (%)	9	8	11
EVR (%)	68	65	63
VR at end of treatment (%)	46	31	53
SVR (%)	29	19	49
Relapse rate (%)	36	40	8

WBR, standard weight-based ribavirin (∼13.3 mg/kg/day); HDR, high-dose weight-based ribavirin (∼15.2 mg/kg/day); VR, virological response (HCV RNA undetectable); RVR, rapid virological response (VR at 4 weeks); EVR, early virological response (VR at 12 weeks).

It has been suggested that the use of erythropoietin may be an appropriate strategy for managing anaemia, improving quality of life and increasing adherence to therapy, especially in patients with genotype 1 infection [63]. However, its use was not permitted in registration trials of peginterferons and RBV and no recommendation for its use in anaemia associated with RBV is included in the Summary of Product Characteristics. Moreover, its addition to the treatment regimen would be associated with additional costs, inconvenience and potential side effects [64]. In conclusion, the limited data available concerning use of erythropoietin are insufficient to make clear recommendations.

If shortening treatment below the standard duration is to be considered, careful reassessment of RBV dose is necessary, since RBV dose and treatment duration appear to be closely linked. In a prospective Austrian study, reducing the dose of RBV to 400 mg did not adversely affect the rate of SVR compared with the standard 800 mg daily dose in genotype 2 and 3 infected patients treated for 24 weeks [65]. However, due to the limited data available, further studies in RBV dose and treatment duration are warranted before any recommendations can be made. In our opinion, weight-based dosing of RBV is advantageous for genotype 1-infected patients, while its relevance for genotype 2 and 3 infected patients remains to be further elucidated, particularly for shorter treatment duration and for patients without RVR. Generally and independent of HCV genotype, RBV dose is less important in patients with RVR and becomes more and more important if only cEVR or slow response is present. The higher percentage of patients with genotype 2 and 3 achieving RVR in comparison with genotype 1 explains the observed differences between genotypes.

#### The influence of adherence/dose reduction on sustained virological response

Both adherence and RBV dose have a major impact on SVR rates. Patients who receive the optimal dose of peginterferon and RBV for the planned duration have higher rates of SVR than those who require dose reductions [21,66–68]. Adherence to therapy is important for treatment success, especially in patients with genotype 1 infection who undergo longer-term treatment, as there is evidence that extended therapy may improve SVR in some patients. Adherence during the initial treatment period is especially important, as early viral suppression is a positive predictor of SVR. Therefore, adherence during the early stages of treatment may be more crucial than overall adherence [22].

Given these findings, physicians should discuss the importance of adherence with patients before initiating therapy. Education of patients, family members and caregivers about potential side effects and their prospective management is an integral aspect of treatment. Frequent monitoring of patients to assess their neuropsychiatric health and social functioning, as well as the clinical side effects of HCV therapy, are important aspects of patient management.

The rapid and effective management of side effects is crucial for treatment success, as adverse events such as anaemia, negatively affect adherence. Identifying and addressing the main side effects of HCV therapy can therefore improve adherence to treatment and potentially allow optimal outcomes to be achieved [69]. Dose reductions are used to manage adverse events encountered during therapy. A recent retrospective study of 569 patients enrolled in phase III trials of 48 weeks’ treatment with peginterferon alfa-2a and RBV, showed that reductions in RBV (≤97% cumulative dose) were more frequent than those of peginterferon alfa-2a (43%*vs* 27%) [70]. Neither EVR nor SVR were affected adversely by RBV reductions when the cumulative RBV exposure was greater than 60%. However, SVR was reduced significantly (*P*=0.0006) in patients with less than 60% cumulative RBV dose. Currently, guidelines suggest that levels of RBV should be reduced to 600 mg, qd, in cases of anaemia (<10 g/dL), which could reduce cumulative RBV levels below the 60% threshold in genotype 1 patients. It has been suggested that more gradual incremental dose reductions of 200 mg may be less likely to impact on SVR than the *ad hoc* reduction to 600 mg, particularly in patients infected with genotype 1 where RBV dose appears to have a greater impact on SVR compared to other genotypes [10]. Prospective studies, however, would be required to establish more clearly the impact of RBV dose reduction on SVR.

### Re-treatment of patients

A major problem for physicians managing patients with CHC is patients with an end-of-treatment virological response but no SVR (relapsers) or patients with HCV RNA detectable at end of treatment with current standard therapy (non-responders). Re-treatment of relapsers is more likely to yield favourable results than re-treatment of non-responders. Re-treatment with peginterferon plus RBV has been investigated in patients who relapsed after interferon monotherapy or interferon plus RBV therapy. Patients who have relapsed following treatment with standard interferon-based regimens often respond to re-treatment with peginterferon plus RBV. In these patients, SVR rates of 41–59% have been reported. Response to re-treatment is most likely in non-genotype 1 patients, patients with mild or moderate fibrosis, and patients with low viral load at baseline [71–77]. Peginterferon plus RBV re-treatment should therefore be considered for all patients who have previously responded to a conventional interferon-based regimen and subsequently relapsed.

Treatment failure can be related to tolerability problems and subsequent discontinuation. It is conceivable that patients non-responsive to one form of peginterferon may be more responsive to treatment with the other form of peginterferon. Furthermore, additional efforts to manage side effects in patients retreated with peginterferon plus RBV may improve adherence and so improve chances of achieving an SVR.

Treatment failure is associated with a higher long-term mortality [78]. Data suggest that selected patients who fail to achieve SVR may benefit from re-treatment with peginterferon-based regimens; however, rates of SVR following re-treatment are far lower than those achieved in treatment-naïve patients. Overall, SVR rates of 4–26% have been reported, with patients who failed to respond to standard IFN monotherapy being most likely to respond to re-treatment with peginterferon plus RBV [71–76,79,80]. Following the results of the ongoing EPIC-3 trial, peginterferon alfa-2b in combination with RBV was recently approved for the re-treatment of relapsers or non-responders to *a prior* course of interferon alfa (pegylated or non-pegylated) with RBV. In this trial, 1336 patients with moderate to severe fibrosis who failed previous interferon-based treatment received peginterferon alfa-2b plus RBV for up to 48 weeks and were followed for a further 24 weeks. 23% achieved SVR. The authors found a strong correlation between achieving SVR and achieving a cEVR with undetectable HCV RNA levels at week 12 of treatment [81]. In the REPEAT study, which investigated the effects of intensified treatment with higher fixed-dose induction of peginterferon and/or longer treatment duration in previous non-responders to peginterferon alfa-2b plus RBV, re-treatment with fixed-dose induction and longer duration provided the highest SVR rates and the lowest relapse rates [82].

While some patients are classified as virological relapsers/non-responders, they may have a biochemical response – i.e. reduction or normalization of ALT. Preliminary results from the HALT-C trial showed that peginterferon alfa-2a maintenance therapy led to improvements in ALT level, HCV viral load and necroinflammation. Despite this however, long term maintenance therapy did not show any effect on the rate of disease progression [28]. These findings were in accordance with a long-term study of peginterferon alfa-2b in non-responders which found no difference in the rate of serious long-term liver complications including decompensation and hepatocellular carcinoma despite improvements in fibrosis [83]. In the long-term COPILOT study comparing colchine with low-dose peginterferon alfa-2b, improved disease free survival associated with peginterferon alfa-2b maintenance therapy occurred almost exclusively in patients with portal hypertension [26]. Given these data, the role of long-term, continuous therapy with peginterferon (or RBV or both) for non-responders cannot be generally recommended.

In summary, decisions regarding re-treatment should include consideration of the severity of the underlying liver disease, adherence/compliance and tolerance issues, the previous therapy and type of response to it, viral genotype and other predictive factors for response [4].

## Conclusion

In conclusion, recent research suggests a number of potential approaches for optimizing therapy for patients with CHC and, as a consequence, increasing SVR rates. Here we have attempted to analyse these findings and translate them into practical advice, to help practitioners make evidence-based treatment decisions in everyday clinical practice. We have also highlighted areas where currently available data do not provide conclusive evidence to suggest amending treatment approaches at present. There is potential for further individualization of therapy when such data become available.

## Abbreviations:

ALT: alanine-aminotransferase
cEVR: complete early virologic response
CHC: chronic hepatitis C
EOT: end-of-treatment
HCV: hepatitis C virus
LVL: low-viral load
pEVR: partial early virologic response
RBV: ribavirin
RVR: rapid virological response
SVR: sustained virological response
